# Can Physical Prehabilitation Reverse Frailty in Elderly Patients Before Spine Surgery? A Case Report

**DOI:** 10.7759/cureus.84465

**Published:** 2025-05-20

**Authors:** Regina Knudsen, Nicholas Ma, Keri Ann Markut, Basma Mohamed

**Affiliations:** 1 Anesthesiology, University of Florida College of Medicine, Gainesville, USA; 2 Orthopedic Surgery and Sports Medicine, University of Florida College of Medicine, Gainesville, USA; 3 Anesthesiology, Duke University, Durham, USA

**Keywords:** frailty, geriatric medicine, optimization, patient optimization, physical prehabilitation, spine surgery

## Abstract

Frailty is a syndrome that results from age-associated decline of physiological function and decreased response to stressors. It has been associated with postoperative adverse events in the spine surgery population. Evaluation of frailty in older adults undergoing surgery is becoming increasingly incorporated in the preoperative evaluation due to the growing number of aging patients requiring surgery. However, which optimization strategies should be incorporated into the preoperative plan to improve the patient’s overall health and quality of life is unclear. Physical prehabilitation has been evaluated in the spine surgery population. However, prehabilitation before spine surgery has mainly focused on cognitive behavioral therapy and physical exercise to alleviate pain. None of the current studies for prehabilitation in spine surgery addressed the role of physical prehabilitation in reversing frailty in older adults. This case report presents the impact of physical prehabilitation on frailty indices and immediate postoperative outcomes in frail spine surgery patients. We present two patients who participated in a physical prehabilitation program for frail older adults requiring spine surgery. Both patients had significant improvement in their frailty, functional capacity, and overall quality of life, as indicated subjectively by the patients. One patient opted out of surgery due to overall improved functional capacity. In this report, we also highlight the impact of physical prehabilitation on the possibility of reversing frailty. Frailty is a well-known risk factor for postoperative adverse events after spine surgery, and whether to modify this risk factor to improve outcomes is still a question that requires future well-designed research studies. This case report shows that physical prehabilitation is a feasible intervention to reverse or prevent the worsening of frailty in spine surgery patients.

## Introduction

An increasing number of elderly patients present with symptomatic degenerative spine disease and adult spine deformity requiring complex spine surgery [1]. Performing spine surgery for older adults might result in an increased risk of postoperative adverse events due to the complexity of their medical comorbidities and poor functional and cognitive status associated with chronic back pain. Prolonged hospitalization and discharge to a skilled nursing facility contribute to the increased economic burden and utilization of healthcare resources.

Frailty syndrome is a better predictor of postoperative adverse outcomes compared to age. A higher frailty score is correlated with an increased risk of postoperative complications and mortality [2]. Despite the increased risk of postoperative adverse events in frail older adults, preoperative optimization is not routinely offered in clinical practice to improve patients’ outcomes due to the lack of standardization of frailty assessments and the heterogeneity of implementing prehabilitation programs for frail patients [3]. There continue to be gaps in the existing literature regarding specific preoperative optimization interventions that may reverse frailty and improve patients’ functional status. Reversing frailty and optimizing the quality of life, including functional status, may improve postoperative outcomes. Prehabilitation has been studied in the frail general surgery population and has been shown to improve functional status and a patient’s potential for postoperative rehabilitation [4-8]; however, prehabilitation has not been evaluated in frail elderly patients undergoing spine surgery. Permissions from these patients were obtained to publish this report. Our case report follows the EQUATOR publishing guidelines for case reports.

This case report was previously presented as a meeting abstract at the University of Florida College of Medicine’s Annual Celebration of Research on February 25, 2020.

## Case presentation

Case 1

A woman in her 70s with lumbosacral radiculopathy and adult spine deformity presented for elective, staged, complex spine surgery consisting of anterior lumbar interbody fusion (ALIF) at levels L4-L5 and L5-S1, followed by posterior spinal fusion. Her medical history was significant for hypertension and compensated congestive heart failure. Additional preoperative risk factors included her age and the associated comorbidities, both optimized and stable. Per our institutional spine center protocol, all patients should achieve smoking cessation for a minimum of eight weeks before elective spine surgery. All geriatric patients (65 and older) will receive the Mini-Cog test, and if positive, they will proceed with extensive neuropsychological testing.

Upon evaluating her functional capacity using the Fried Frailty Index, her score was 4 out of 5, attributed to exhaustion, weak grip strength, slow walking speed, and low physical activity. The patient was offered physical prehabilitation before the ALIF procedure; however, she refused due to the severity of her back pain and her belief that surgery was the only solution to “fix” her back pain and allow her to participate in physical therapy. Her postoperative phase after the ALIF was complicated by an unexpected stay in the ICU for 24 hours, followed by an eight-day hospital stay and discharge to an inpatient rehabilitation facility. She was readmitted within 30 days with wound complications. In preparation for her stage 2 posterior spinal fusion surgery, her functional status deteriorated despite her stay in rehabilitation for two months. Using the Fried Frailty Index, her score was 5 out of 5. Her modified Frailty Index-11 (mFI-11) was 27%, indicating she was frail. A six-minute walk test (6MWT) to further assess her functional capacity showed 8% predicted (30 meters). This time, the patient agreed to physical prehabilitation prior to proceeding with stage 2.

**Figure 1:**
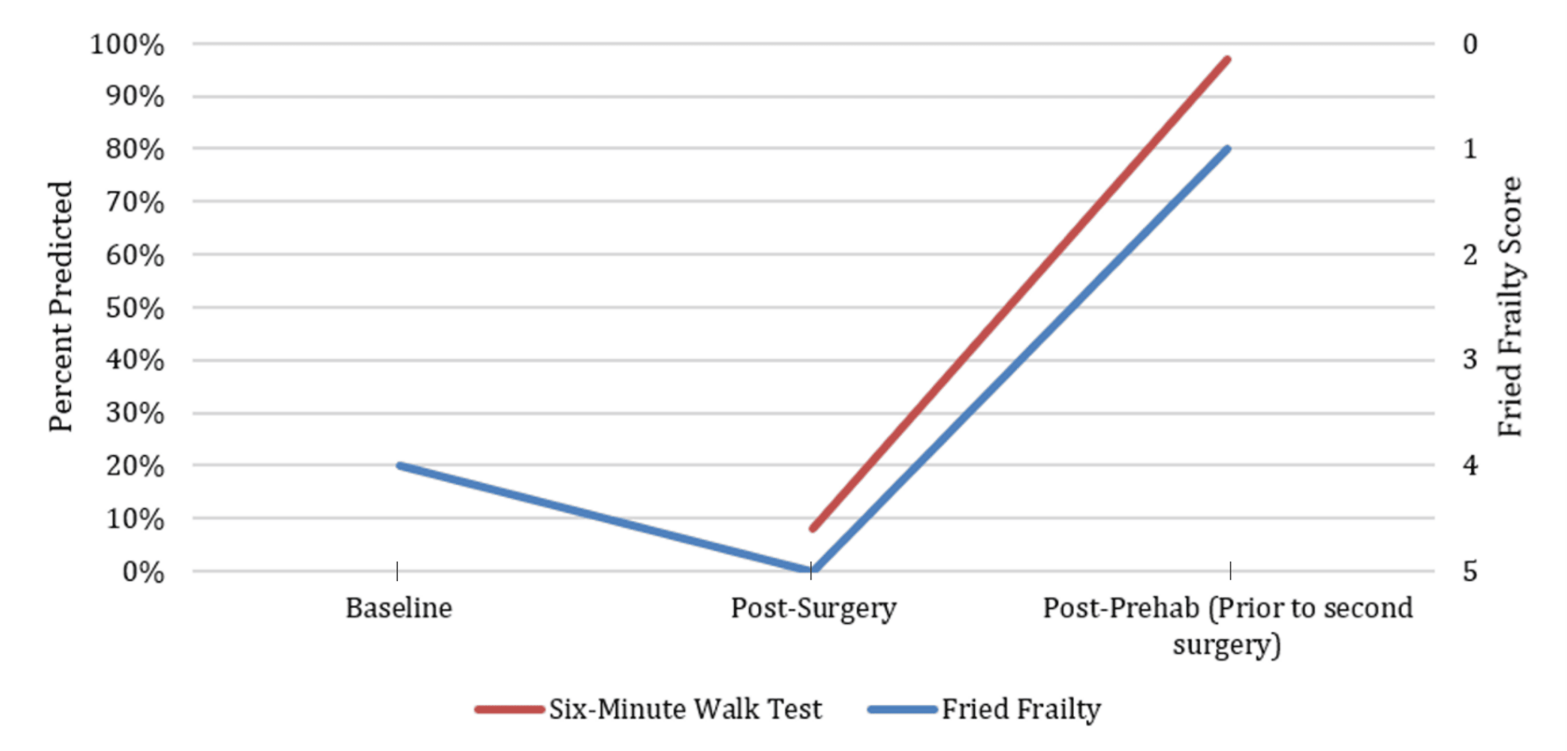
Case 1: Functional assessment at baseline, post-surgery, and prior to the second scheduled surgery After declining prehabilitation prior to her first surgery, the patient decided to complete 12 weeks of prehabilitation prior to her second surgery, which demonstrated an improvement in frailty score and 6MWT. These significant improvements led to the decision not to proceed with the second surgery. 6MWT, six-minute walk test

After completing 12 weeks of physical therapy (Figure 1), her repeat Fried Frailty score was 1 out of 5, attributed to weak hand grip, and her 6MWT improved to 97% predicted (358 meters). Her functional capacity showed significant improvement compared to her initial evaluation prior to the first surgery. She could now walk 0.5 to 1 mile daily and resumed activities of daily living, such as shopping and light housekeeping chores. She reported significant improvement in energy level and overall sense of well-being. After discussion, the patient and her neurosurgeon agreed that her current state had dramatically improved, and they opted not to proceed with surgery after reviewing her symptoms, current quality of life, and lumbar spine imaging.

Case 2

A woman in her 60s with thoracic stenosis presented for elective thoracolumbar spine surgery (T12-L5 laminectomy and T12-L3 fusion). Her main symptoms included neurogenic claudication due to spinal stenosis. Her medical history was significant for morbid obesity (BMI = 42 kg/m²) and osteoarthritis. Upon evaluating her functional capacity using the Fried Frailty Index, her score was 3 out of 5, attributed to exhaustion, slow walking speed, and low physical activity. Her mFI-11 score was 0 out of 1, indicating she was not frail. A 6MWT was used to further assess her functional capacity, which showed 35% predicted (134 meters).

**Figure 2:**
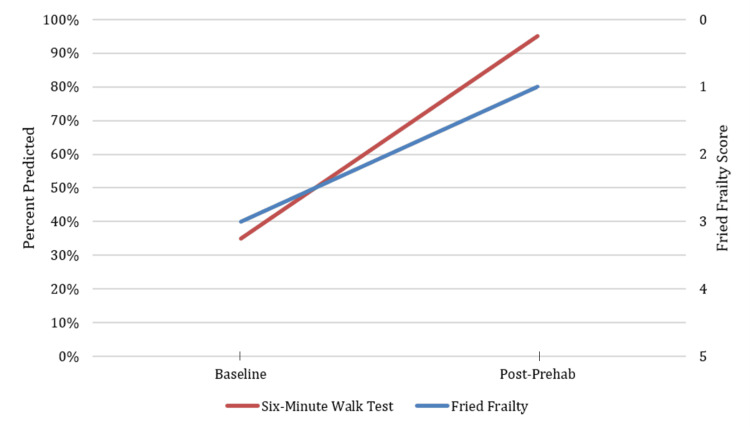
Case 2: Functional assessment before and after completing 12 weeks of prehabilitation showing an improvement in frailty score and 6MWT prior to surgery 6MWT, six-minute walk test

After completing 12 weeks of physical therapy (Figure 2), her repeat Fried Frailty score was 1 out of 5, attributed to exhaustion, and her 6MWT distance improved to 95% predicted (362 meters). Her perioperative course was uneventful, with standard anesthesia induction and maintenance. Her perioperative pain management included an intraoperative dose of methadone at 0.2 mg/kg and fentanyl. Her postoperative stay was uneventful, and she was discharged home with home physical therapy after five days of hospital stay. The patient did not experience any postoperative complications or readmission within 30 days after surgery.

## Discussion

**Figure 3:**
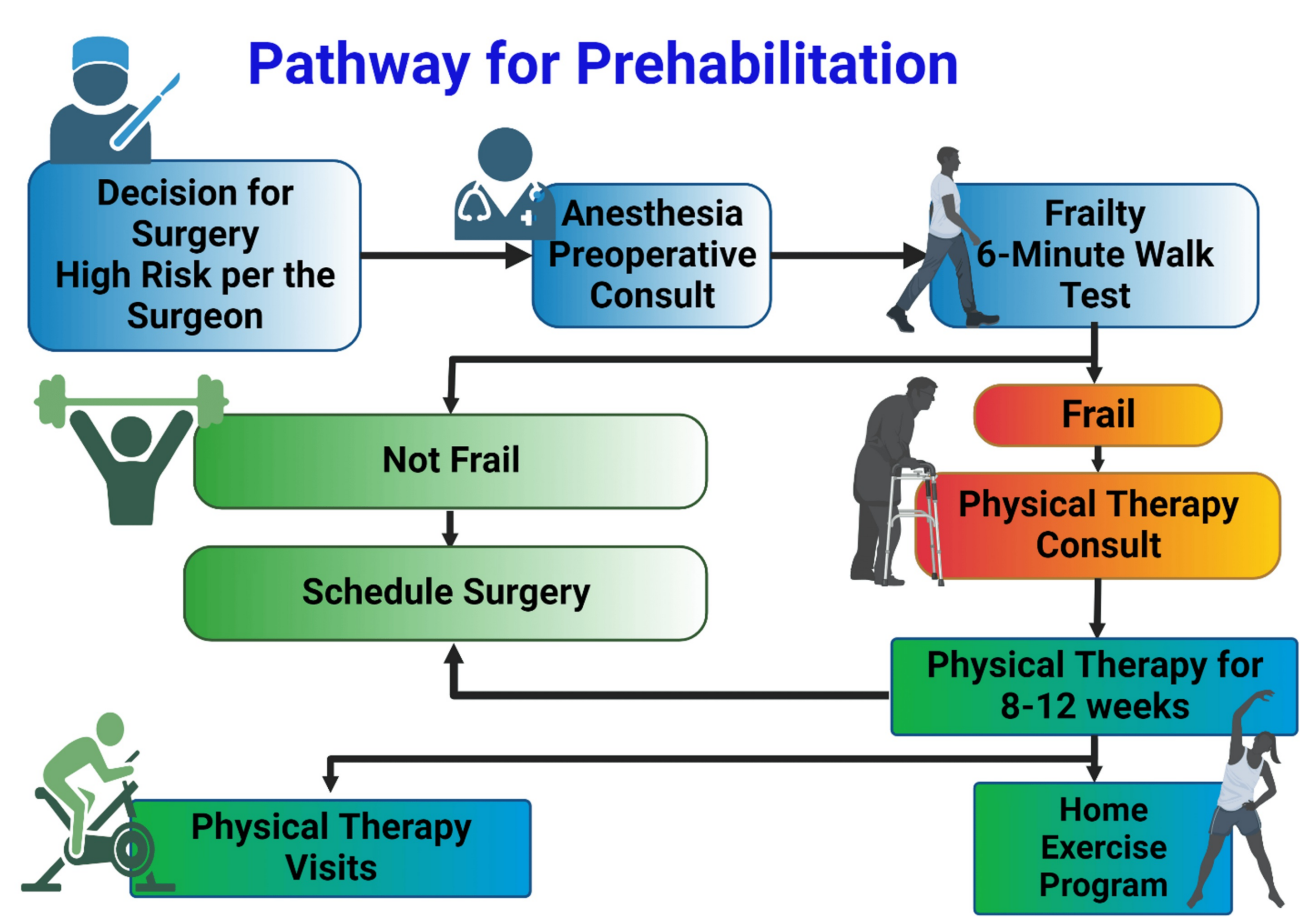
Preoperative physical prehabilitation pathway for frail older adults undergoing spine surgery Created by Basma Mohamed using BioRender (2025). https://BioRender.com/svi10bk

Frailty syndrome in the elderly population is a predictor for worse postoperative outcomes, including prolonged length of hospital stay, postoperative complications, and possibly increased risk of mortality after surgery [9,10]. Elderly patients with degenerative spine disease are at increased risk of developing frailty due to associated comorbidities and the chronicity of their back and neck pain before requiring surgery. Frailty assessment tools have been created, validated, and studied in the spine literature with a focus on tailoring different assessment tools to assess the risk of different spine pathologies precisely. Moskven et al. identified 14 frailty assessment tools utilized in the surgical spine population [10]. Of those tools, the Fried Frailty Index is sensitive to evaluating the trajectory of frailty and the impact of frailty optimization strategies on the severity of frailty [10]. This case report shows that physical prehabilitation can reverse frailty and improve patients’ functional capacity, as shown by the improvements in the 6MWT and their overall quality of life, as indicated subjectively by the patients. As noted with Case 1, general physical therapy programs that do not target frailty may not give the same results. A program tailored toward strength training, mobility, and endurance is the key to a structured prehabilitation program. Our institutional physical therapy program (Figure 3) aims to focus on frail older adults undergoing spine surgery and to identify impairments or safety issues, assess physical performance, and develop a therapy plan that includes a home exercise program targeting core muscle strength, helping patients achieve independence in mobility and transfers, and educating them on postoperative expectations and postsurgical precautions.

Few case reports evaluated the impact of prehabilitation on optimizing frailty (Table 1) [4-8,11]. Villers et al. evaluated the impact of physical prehabilitation in a patient undergoing reverse total shoulder replacement and found that undergoing physical prehabilitation may delay the need for surgery altogether [5]. Carli et al. evaluated the impact of multimodal prehabilitation in an octogenarian patient undergoing robotic-assisted total abdominal hysterectomy and found that prehabilitation may have prevented further episodes of postoperative delirium [12].

**Table 1:** Prehabilitation case reports in spine vs. non-spine patients 2MWT, two-minute walk test; 5TSST, five times sit to stand test; 6MWT, six-minute walk test; DASI, Duke Activity Status Index; HEP, home exercise program; HGS, hand grip strength; LOS, length of hospital stay; NPRS, Numeric Pain Rating Scale; ODI, Oswestry Disability Index; PROMIS-29, Patient-Reported Outcomes Measurement Information System; PPPD, pylorus-preserving pancreaticoduodenectomy; QuickDASH, Quick Disabilities of Arm, Shoulder and Hand; RBANS, Repeatable Battery for the Assessment of Neuropsychological Status; SF-36, 36-Item Short Form Health Survey; SPADI, Shoulder Pain and Disability Index; SRT, steep ramp test; STS, sit-to-stand test; TKA, total knee arthroplasty; TUG, Timed Up and Go Test; VSAQ, Veterans Specific Activity Questionnaire; WOMAC, Western Ontario and McMaster Universities Index

References	Patient age/sex	Preoperative frailty score collected (Y/N)	Preoperative assessment of frailty functional status	Surgery	Preoperative physical prehabilitation	Other prehab modalities (cognitive, nutrition) (Y/N)	Functional outcome measures	Clinical outcome measures	Implications	Future directions
Brown et al. (2020) [4]	56 M	N	N/A	Heart transplantation	Supervised, 40-60 min, 4×/week, 4 weeks	N	Tolerance for activity, metabolic equivalent of task	Discharge disposition	Initiation of inpatient cardiac prehab may help recovery and physical independence prior to enrollment in phase II cardiac rehab at discharge	Not highlighted in the published case report
Villers et al. (2020) [5]	62 M	N	NRPS, SPADI, QuickDASH	Reverse total shoulder replacement	HEP, 6 sessions over 32 days	N	NPRS, SPADI, QuickDASH	Pain, joint mobility, muscle strength, and function	A physical therapy prehab program may delay the need for surgery	Not highlighted in the published case report
van Beijsterveld et al. (2021) [7]	75 F	N	SRT, HGS, TUG, 2MWT, 5TSST, VSAQ, DASI, nutritional assessment	PPPD	Partly supervised, 60 min, 2×/week, 4 weeks	Y, Cognitive	HGS, 2MWT, 5TSST	Discharge disposition	Preop risk assessment can support clinical decision-making in coalition with a high-risk patient opting for major abdominal surgery	Not highlighted in the published case report
Brown et al. (2010) [8]	69 F	N	6MWT, STS, time to ascend and descend two flights of 11 stairs each, WOMAC	TKA	HEP, 60 min, 3×/week, 4 weeks	N	6MWT, STS, time to ascend and descend two flights of 11 stairs each, WOMAC	Knee strength, pain	Prehab improves functional capacity up to 30%	Prehab might be effective at facilitating rehab following a TKA
Knudsen et al. (2023) [11]	83 F	Y, Fried Frailty score 4/5	6MWT, Fried Frailty Index, SF-36, ODI, PROMIS-29	L1-L5 multilevel fusion	Supervised, 60 min, 2-3×/week, 8 weeks	Y, Nutrition and cognitive	6MWT, Fried Frailty Index, SF-36, ODI, PROMIS-29	Discharge disposition, hospital LOS, home discharge	Multimodal prehab can be a feasible modality for preoperatively optimizing frail older adults undergoing complex spine surgery	Instead of declining surgery, spine surgeons might offer prehab as an optimization intervention for frail older adults with spine disease to improve their functional status
Carli et al. (2012) [12]	88 F	N	6MWT, SF-36, nutritional assessment (albumin), RBANS	Robotic-assisted total abdominal hysterectomy	HEP, 60 min, 3×/week, 3 weeks	Y, Nutrition and cognitive	6MWT, SF-36, nutritional assessment (albumin), RBANS	Tasks assessing attention	Prehab optimized the health of this patient and may have prevented further episodes of postop delirium	Prehab clinical trials

Few studies evaluated the impact of optimizing frailty on postoperative outcomes after spine surgery. Yagi et al. aimed to evaluate the impact of treating frailty, measured through a deficit-accumulation index, on postoperative clinical outcomes in patients undergoing adult spine deformity surgery [13]. In this retrospective multicenter database review, the authors found that despite appropriate treatment for each comorbidity listed in the mFI-11, according to current guidelines, frailty continued to be associated with increased risk of postoperative complications and poor postoperative outcomes after adult spine deformity surgery. Different confounders, including the invasiveness of surgery, might explain these findings. In a systematic review, Janssen et al. evaluated the current prehabilitation programs and their impact on perioperative outcomes in spine surgery [9]. Due to the heterogeneity of the included studies, the authors could not pool results from physical prehabilitation programs. Their analysis focused on the impact of cognitive behavioral therapy as a prehabilitation program on different perioperative outcomes, including physical functioning, back and leg pain, health-related quality of life, and psychological outcomes such as anxiety and depression, as well as hospital length of stay and analgesic use. They concluded that existing prehabilitation programs in the form of cognitive behavioral therapy did not impact perioperative outcomes. None of the included studies focused on frail patients undergoing spine surgery. To date, none of the existing studies that proposed prehabilitation as a preoperative optimization intervention focused on reversing frailty, targeted frail patients, or evaluated the impact of reversing frailty on patients’ overall functional capacity, quality of life, or immediate or long-term postoperative outcomes after spine surgery [14-20].

There are several limitations to our approach. We implemented physical prehabilitation as a quality improvement preoperative optimization project to evaluate the impact of prehabilitation. Patient-reported and long-term postoperative outcomes were not formally evaluated, such as the Oswestry Disability Index or other quality-of-life measures like the Medical Outcomes 36-Item Short Form Health Survey or EuroQol 5-level EQ-5D version. We also did not evaluate the difference in the mFI-11 score before and after prehabilitation. Per the preoperative calculation, the mFI-11 score was inconsistent in both patients. In addition, based on the comorbidities, the mFI-11 score may not change. The mFI-11 has not been a sensitive tool for evaluating the frailty trajectory after interventions [10]. Our preoperative optimization pathway emphasizes the optimization of medical comorbidities but is unlikely to alter the mFI-11 score. Additionally, both patients showed adult spine deformity as the main spine pathology; however, we could not use the adult spine deformity frailty index, a 40-variable-long measure, as it was deemed not clinically practical to use routinely at the preoperative anesthesia clinic. Finally, the Fried Frailty Index has been studied in the spine and general surgery populations; however, it has not yet been validated in spine surgery patients. Meanwhile, the Fried Frailty variables are easy to assess and show the potential for reversibility, as shown in this case report, which can be an outcome for improvement in future studies in addition to the 6MWT.

## Conclusions

To our knowledge, this is one of the first case reports to highlight the impact of physical prehabilitation on frailty indices and to show the impact of physical prehabilitation on immediate postoperative outcomes in frail spine surgery patients. Prehabilitation can potentially optimize frailty and improve patients’ quality of life, which is the ultimate goal of spine surgery for degenerative spine disease. Physical rehabilitation has been evaluated in the frail general surgery population and has been shown to improve frail patients’ preoperative functional capacity and their potential for postoperative rehabilitation. However, the current literature does not evaluate the role of physical prehabilitation in reversing frailty indices to improve outcomes in general or in spine surgery patients. Well-designed research studies are needed to evaluate the potential for physical or multimodal prehabilitation in the preoperative optimization of frail older adults undergoing spine surgery. Physical prehabilitation might be the hope for this vulnerable patient population, not only to prepare them to cope with the stress of surgery but also to optimize their overall functional capacity and ability to participate in postoperative rehabilitation, minimize the risk of complications, and improve their overall quality of life.
